# Local Organizations Supporting Implementation of Graphic Health Warnings for Tobacco in Underserved Communities: A Qualitative Inquiry

**DOI:** 10.3389/fpubh.2018.00322

**Published:** 2018-11-13

**Authors:** Shoba Ramanadhan, Rebekah H. Nagler, Jaclyn M. Alexander-Molloy, Kasisomayajula Viswanath

**Affiliations:** ^1^Center for Community-Based Research, Dana-Farber Cancer Institute, Boston, MA, United States; ^2^Department of Social and Behavioral Sciences, Harvard T.H. Chan School of Public Health, Boston, MA, United States; ^3^Hubbard School of Journalism & Mass Communication, University of Minnesota, Minneapolis, MN, United States; ^4^School of Nursing, University of Massachusetts—Boston, Boston, MA, United States

**Keywords:** graphic health warnings, community-based organizations, implementation science, community-level, tobacco

## Abstract

**Background:** Graphic health warnings (GHWs) on cigarette packages offer the potential to support tobacco cessation and prevention of initiation. Guidance for supporting implementation at the local level is limited, which can lead to missed opportunities to amplify the health impact of GHWs. This study examines the potential for local organizations engaged in tobacco control in underserved communities to support GHW implementation.

**Materials and Methods:** Key informant interviews were conducted with 20 leaders in the three partner communities of Boston, Lawrence, and Worcester, Massachusetts. Data were analyzed using a combination of inductive and deductive methods, grounded in a framework analysis approach.

**Results:** First, participants expected local organizations to play a diverse range of roles to support GHW policy implementation, ranging from convening local actors to offering complementary health education activities. Second, there is a need for external agencies to actively support local organizations during the pre-implementation and implementation phases, e.g., by engaging local organizations and providing resources and technical assistance. Finally, participants noted concerns about potential disconnects between the GHWs and the needs of underserved populations.

**Discussion:** With the necessary supports, local community organizations can be important implementation partners to maximize the impact of GHWs and ensure that benefits accrue to members of underserved communities.

## Introduction

The World Health Organization (WHO) put forth the Framework Convention on Tobacco Control to address the health impact of tobacco use, which is expected to cause over one billion premature deaths in the twenty-first century. This global public health treaty—the first of its kind—leverages the rich tobacco control evidence base to promote recommendations to address demand- and supply-side reduction measures and environmental protection, among other targets. One demand-reduction strategy is the use of graphic health warnings (GHWs), labels that cover 30–50% of the cover of a cigarette package and relay information regarding the consequences of tobacco use, often including pictures (1). GHWs have been shown to attract smokers' attention, increase awareness of smoking-related health risks, decrease cigarette consumption, motivate cessation attempts, increase use of cessation services, and increase abstinence (2–6), including among low socioeconomic position (SEP) groups and racial/ethnic minority populations (7, 8).

In the case of GHWs, the WHO offers guidelines for implementation of health warnings on tobacco packages (9), but these strategies emphasize actions for national or state government or large agencies, such as decisions about the size, placement, or content of the messages. While the guidelines highlight the utility of supporting campaigns, details are limited and typically overlook contributions local organizations can make. The local level, a diverse set of jurisdictions including counties and cities as well as school districts and neighborhoods, has a great impact on the success or failure of policy implementation (10, 11). After all, local implementers must have sufficient capacity, motivation, and resources to support the policy (12–14). Local actors must also be able to leverage their knowledge of local context to support broader policy implementation goals (12, 15). A recent review identified key supports for policy development and program implementation at the community level, including collaboration and systems approaches, consensus regarding goals and objectives (including the definition of roles and ownership), local planning and activity to incorporate context and increase sustainability, and leadership and guidance to support action (16). Related to GHWs, local action might include supporting policy enforcement, such as enforcing display requirements for GHWs. Another set of activities is broader, including addressing the increased demand for cessation services and leveraging opportunities to engage in health promotion to take full advantage of GHWs (2–4, 17, 18).

The capacity for local organizations to support GHWs is of particular significance in communities with many members of low SEP, who are disproportionately affected by smoking (19, 20). Local organizations may be able to buffer the creation or exacerbation of inequalities that sometimes occur when population-level campaigns have a greater positive impact on higher SEP groups exposed to the same information as low SEP groups (21). Thus, the study of local organizations in low SEP communities affords an opportunity to assess potential *communication inequalities*, or differences among social groups in the generation, manipulation, and distribution of information at the group level and differences in access to and ability to take advantage of information at the individual level. This theoretical perspective prompts attention to communication inequalities as potential mediators of the relationship between social determinants and health outcomes, thus serving as a partial explanation for health disparities (22, 23).

The success of GHWs will depend on implementation efforts at multiple levels; understanding the necessary supports for local implementation is critical for success. With this study, we explore what it means for local organizations to support the implementation of GHWs, with an emphasis on barriers to and facilitators of such supports. Qualitative methods were ideal for this exploration of a wide range of contextual and system factors that may affect implementation. Our goals for this study were contextual (focused on understanding the characteristics of what already exists in terms of attitudes, experiences, needs, and key components of the system) and strategic (to generate opportunities to improve systems) (24).

## Materials and methods

### Project overview

Data for this study come from Project CLEAR, an assessment of the impact of GHWs on underserved populations. To complement the study team's assessments of the impact of GHWs on community members (25–27), this study focuses on community-level policy implementation. The study was conducted in three partner communities: Boston, Lawrence, and Worcester, MA, with support from longstanding partners representing health coalitions. The three communities are diverse in terms of size and sociodemographic composition, and each included significant numbers of individuals from racial/ethnic groups or socioeconomic groups experiencing health disparities across a range of indicators (28). Census data from 2010 (29) are presented in Table 1, highlighting this diversity. Each of these communities has a vibrant health sector, with progressive, multi-sector efforts to address health disparities and the social determinants of health.

**Table 1:** Sociodemographic characteristics of partner communities.

	**Boston**	**Worcester**	**Lawrence**
Population	602,000	180,000	76,000
Race/ethnicity (%)			
White, non-Hispanic	48	64	22
Black, non-Hispanic	23	9	1
Asian, non-Hispanic	9	6	3
Hispanic (any race)	17	20	73
Percent living below the poverty line	22	22	28

### Study design and participants

We conducted semi-structured key informant interviews with a purposeful sample of local leaders in tobacco control in each of the three communities. We utilized a combination of positional and reputational sampling approaches (30), starting with an individual in each community identified by community partners and local outreach staff as occupying a position of leadership in the area of tobacco control. Additionally, each participant was asked to nominate other local leaders in tobacco control (the reputational portion of the sampling approach). We selected additional potential participants based on nominations and with the goal of ensuring representation across sectors (healthcare, government, and community) and diversity of population groups served, prioritizing individuals working with underserved groups. The initial set of participants were familiar with some or all of the research team members, but subsequent participants did not necessarily have a connection to the team.

We recruited potential participants by phone and email. Individuals who agreed to participate were sent materials in advance of the interview, including consent documents and pictures of the nine GHWs proposed in 2011. The interviews were conducted by phone by a cultural anthropologist, an experienced interviewer and long-time collaborator with the authors. We chose to utilize telephone-based interviews (vs. in-person interviews) to reduce the burden to participants. Given that participants were discussing a non-sensitive topic and were accustomed to publicly sharing their opinions as part of their leadership roles, phone-based interviews were seen as an appropriate channel by which to reduce participant burden. Interviews took about 1 h.

The interviews were audio-taped and were transcribed by an independent, professional transcriptionist. We stopped conducting interviews when we reached theoretical saturation (the point at which interviews were no longer yielding new themes) (31); this occurred after 20 interviews. Two members of the study team (including SR) reviewed transcripts after each interview to determine which nominations to accept, to refine prompts and probes, and to determine the point at which theoretical saturation was reached. The Harvard University Institutional Review Board approved the research procedures.

Informed by the Communication Inequalities theoretical perspective, we developed an interview guide that explored the needs of local organizations working with underserved populations that often experience challenges in taking full advantage of health promotion innovations. Rich description of both the context in which GHWs would be implemented and identification of opportunities to improve supports is expected to offer buffers to the creation or exacerbation of new communication inequalities. Thus, the interview guide focused on how local organizations would support GHW implementation, potential barriers to successful implementation, the impact of city/town support on efforts to support GHWs, recent experiences with policy implementation, and identification of key players for local implementation.

### Data analysis

Two researchers (SR and JAM) used a two-stage coding process that included both prefigured and emergent codes. Initial coding was primarily descriptive, with questions from the interview guide serving as the framework for the prefigured coding structure. With an orientation toward framework analysis (32, 33), the second phase of coding used a more inductive approach. Categories that emerged from the data formed the broader thematic framework, which was then applied to all transcripts. We selected and presented exemplar quotations for important themes. After drafting initial results and interpretations, we conducted member-checks (34) with community partners as well as a set of practitioners similar to those who would be charged with policy implementation. We refined our interpretations based on member-checks. We utilized NVivo10 (35) to create a single dataset for the project, support the multi-stage dual coding process, and flag exemplar quotations for presentation.

## Results

Twenty key informants were interviewed between November 2014 and June 2015. Most held positions as program directors, physicians, and board members and worked in the public sector (15%), non-profit and community organizations (45%), and healthcare settings (40%). Consistent with the design, an almost equal number of participants were interviewed from Boston (30%), Lawrence (30%), and Worcester (40%). Fourteen of the participants were women.

Three broad themes characterized participants' perspectives regarding local implementation of GHWs. These themes, and the main ways they were expected to manifest, are summarized in Table 2. First, participants described a diverse range of roles for local organizations in supporting implementation of GHWs, from outreach to advocacy and policy enforcement. Second, external assistance (from state and regional agencies) was identified as a critical support, with requests for technical assistance, resources, and stakeholder engagement. Third, participants noted that local organizations may need to buffer potential gaps in utility of GHWs for underserved populations, such as youth, racial/ethnic minorities, those with limited English proficiency, and those of low SEP.

**Table 2:** Key themes (and key manifestations of each) that emerged from the interviews.

**Key theme**	**Key manifestations**
1. Local organizations will need to play a diverse range of roles to support GHW implementation	1a. Health education
	1b. Convening or connecting relevant organizations
	1c. Engaging in policy-making / advocacy / community mobilization
	1d. Delivering healthcare services
	1e. Providing access to community members
2. External assistance will be a critical support for GHW implementation	2a. Early and ongoing stakeholder engagement
	2b. Technical assistance and support from state and federal agencies
	2c. External provision of resources to ensure funding and staff capacity needs are met
3. Local organizations may need to buffer a gap between GHWs and the needs of underserved groups	3a. Youth may react in unexpected and risk-promoting ways to GHWs
	3b. Cultural subgroups and recent immigrants may not connect to / benefit from GHWs
	3c. Persons of low SEP may have competing demands that impact the prioritization of tobacco cessation

### Theme 1: local organizations are expected to play a complementary and diverse set of network roles to support GHW implementation, from conducting health education and outreach to community engagement and advocacy

Almost all participants expected their organizations to support GHWs if/when they are released. When asked to consider other local players that would be critical for GHW implementation, a diverse range of organizations was nominated, and these organizations were expected to play a variety of roles in supporting GHW implementation. In order of decreasing frequency, these roles included health education or program delivery; convening or connecting relevant organizations; engaging in policy-making/advocacy/community mobilization; delivering healthcare services; and providing access to community members, including population subgroups. Other less frequently mentioned roles included educating and engaging stakeholders and playing a regulatory/enforcement role.

#### Health education or program delivery

Participants noted that their organizations would conduct/support health education campaigns to raise awareness about tobacco and GHWs, including sharing information about the quitline and other resources. A range of activities was presented, from posting flyers and posters to conducting presentations/education sessions. Communication campaigns, including the use of social media, were also frequently mentioned.

#### Convening/connecting organizations

Many of the key players nominated as important for local implementation were expected to play a convening or connecting role, such as local coalitions that included large numbers of organizations engaged in health promotion. Participants suggested that the convening/connecting organization could serve as a channel by which to distribute information and coordinate action related to GHWs and tobacco services.

“We're an alliance, so, we do a lot of convening, bringing together.…We have representation both at the local neighborhood level, as well as city-wide organizations and we are a cross-sector alliance. Meaning, we're not just people who focus on health, but, organizations that also focus on social determinants of health—like housing, education, and employment—all of which are important for healthy communities.”—Participant 6

Inter-organizational relationships were noted as an important offset to the scarcity of resources faced by many local organizations. Many participants noted that their organizations would coordinate a response to GHWs in conjunction with other local organizations, thus sharing the burden.

“We would all work together because this information would have to be distributed state-wide.…I would want to make sure if there are local resources, that all the tobacco treatment specialists are aware ahead of time…I think as much preparation as you could do ahead of time, that would be helpful…And, you know, we're not gonna have new resources because these come out…The tobacco program isn't that highly funded…”—Participant 1

#### Policy-making/advocacy/community mobilization

Several organizations were highlighted as important partners in local implementation efforts given their current work with policy creation, advocacy, and community organizing. Many participants mentioned the importance of community mobilization, particularly among underserved and targeted subgroups:

“My guess is we would be excited to mobilize young people to really get the word out about the warning labels…we could engage and educate, like telling policymakers that this is happening, they could skew visibility about it in their communities.”—Participant 10

Participants also highlighted opportunities to leverage the GHW initiative to further broader tobacco control efforts in the community, as in this example:

“We would probably do a report to City Council to talk about this and also use it as, again, as an opportunity to talk about, you know, the issue of how many people in the City of [X] are smoking, who's smoking, what are our compliance rates…and then, use it as an opportunity to, you know, have a community conversation.”—Participant 20

#### Healthcare delivery

Provision of direct services, such as those offered by hospitals and clinics, were highlighted as important roles for implementation, to support abstinence and provide cessation services as needed.

“The majority, I think, of the population of the city comes here for their healthcare, inpatient and outpatient services. We provide education and, and we hold community health fairs…The questions that revolve around healthcare concerns, a lot of them are filtered through us.”—Participant 11

#### Providing access to community members, including population subgroups

The final important role identified was to grant access to community members, particularly those who are not easily reached. Community organizations were expected to reach populations with information and messaging related to tobacco control and leverage trust and connections to provide an entry point for tobacco services. In this way, organizations that do not engage directly in tobacco control can serve as an “informal referral network” and connect community members to useful services.

“It might be obvious, but [churches] provide support to individuals and families in the community, both spiritual and in other ways. Uh, so they're-well, very well-connected with some of our families, and particularly with certain cultural areas. So, if you wanted to target particular cultural groups, our local churches are a good way to do that.”—Participant 18

### Theme 2: resources from and early stakeholder engagement by external agencies will be critical supports

Early stakeholder engagement was highlighted as critical for generating the necessary buy-in required for support of GHWs. Participants noted that stakeholder engagement is not always the norm for policy changes; often organizations are handed an edict and expected to follow it. When discussing a recent local policy change which was not received well, one participant noted:

“I don't know how much education or how much outreach has been done, because there's one thing about telling you, ‘This is the new rule,’ and there's the education piece that says, ‘Okay, this is what's coming down the pike. This is why this should be done this way.”’—Participant 2

Participants also noted that early engagement allows for the development of a coordinated response by local organizations. When asked what could be done to support the implementation of GHWs, one participant noted the following:

“I think making sure that you are having conversations with some of the major players, like you're doing now on the survey with me, and I think it's important not to have communities be blindsided.”—Participant 15

In addition to being a source of early engagement, participants reported a need for technical assistance and guidance from state and federal agencies, such as the Massachusetts Tobacco Cessation and Prevention Program or the FDA. Participants also noted that agencies would need to provide dedicated resources to support this effort, including funding and services. For example, participants mentioned increasing the capacity of the quitline since organizations are not able to provide local resources and would instead refer concerned members of the public to the quitline.

Externally provided resources to address a lack of funding and staff capacity were seen as critical. When discussing the ability to field an outreach campaign, one participant noted the following:

“Doing a letter to the editors, and Tweeting about it and putting it on Facebook and sending out a note to [our constituents]…we have the capacity to do something like that. To do anything bigger, do like a big press release or press conference or something like that, we wouldn't be able to take the lead on it unless we were given resources to do that…”—Participant 13

When discussing state and regional supports, participants also noted a supportive environment for tobacco control at the local and state levels and highlighted the progressive actions taken in their communities and in the state to address tobacco control. They cited examples of support from local boards of health and state-level coalitions and advocacy groups.

### Theme 3: local organizations may need to buffer a gap between GHWs and the needs of underserved groups, such as those of low SEP, young people, and those with limited english proficiency

Many participants were concerned about the fit between GHWs they reviewed and the population subgroups they serve. For example, participants noted that community members who speak a language other than English or recent immigrants might be unable to benefit from GHWs. One participant noted the following as a challenge for supporting GHWs in a diverse community:

“Lots of these health centers have probably eighty-two languages that they know, that they see through their clinics. So, I wanted to share that language is an area of sensitivity and also, culture, humility, and competence, as far as the labels as well, and literacy.”—Participant 15

Another group frequently mentioned as potentially needing additional attention related to GHWs was young people. Participants were concerned that teenagers and young adults might not respond to fear appeals and that the efforts may even be counterproductive. Participants whose organizations work with youth repeatedly noted the importance of engaging youth in anti-tobacco activities as part of implementation activities, partly to ensure the desired effect and partly as a springboard for broader action around tobacco cessation. Participants from organizations that play a convening role in the community, such as the Board of Health and coalition leaders, mentioned youth engagement as an important part of the implementation process to enhance impact.

Participants noted that a major asset for local organizations is the relationships they have with community members. In this way, participants suggested that GHWs might start a conversation around tobacco, but that local organizations would then need to convert that interest into cessation services or appropriate education and prevention activities. Local engagement was necessary for low SEP populations that were expected to have several other competing demands. Local organizations were expected to be able to address tobacco control in the broader context of community members' needs.

## Discussion

The results highlight the potential for local organizations working with low SEP populations to amplify the effects of GHWs and accompanying programming. First, the results highlight how diverse local roles could support the implementation of a national GHW policy. Second, the findings draw attention to the role of external agencies in supporting local organizations during the “pre-implementation” and “implementation” phases, including engagement and provision of resources and technical assistance. Finally, the findings suggest that local organizations can offer wrap-around services or play linking roles to increase the likelihood that GHWs will benefit underserved populations.

First, the findings highlight the potential for diverse local organizations to provide complementary action in support of GHWs. Important roles spanned the health promotion continuum (36), from health education to mobilization and advocacy/policy activities and are consistent with the literature highlighting the importance of community collaboration as an implementation support (16). The convening/connecting function may be of interest from an implementation standpoint given the potential to increase impact and to do so on a limited budget. Coordinated, intersectoral action by diverse partners increases the range of resources available, not only by amassing existing resources but also through creation of new resources (37, 38). As many participants noted, there are limited resources for tobacco control and additional action, including investments for partnerships, will require dedicated resources. This is a worthwhile investment, as a gap in partnership infrastructure has been a major hindrance to local implementation of regional or federal health policies elsewhere (39).

The second important set of findings highlighted the importance of external supports for local organizations to support tobacco control goals. Leadership and guidance are critical supports for program and policy implementation (16), and regional institutions were identified as important potential intermediaries between federal policy and local action. During the preparation phase, external agencies are expected to be critical for activating networks and engaging local organizations to understand and support intended policies. Once implementation starts, external agencies are expected to be important for providing resources, guidance, and technical assistance to support action. These supports, including stakeholder engagement and ongoing feedback and resources, have been identified in the literature as critical to facilitating the application of evidence in practice (15, 40). A similar set of supports was identified as critical for supporting practitioners to counteract tobacco marketing and sales locally at the point of sale. Those practitioners required detailed implementation guidance, capacity-building, and examples from the field (41). For these reasons, relationships and collaborations among community actors, as well as with regional intermediaries, must be maintained and sustained in the long term to support policy implementation. The inputs described by participants highlight the importance of building community capacity—the human capital, social capital, and resources held by local organizations to address pressing health problems and support the well-being of community members (42). For tobacco control, capacity-building could empower local organizations to leverage the informal referral networks they already employ to connect community members with resources.

Finally, local organizations can leverage their deep knowledge of and connections to community members and population subgroups to support underserved populations in accruing the benefits of national policies and accompanying campaigns. As seen in the implementation of evidence-based programs, strategies, and policies, context plays an important role in determining implementation success or failure (15, 43, 44). By leveraging relationships with community members and making the most of their local expertise, local organizations working with the underserved may be able to buffer the differential impact of campaigns that sometimes have less influence on low SEP groups than high SEP groups (21). Local organizations can provide complementary messaging to adapt the messages and address mismatch based on group characteristics, such as language, ethnicity, or migration status (45). Organizations must be equipped with strategies to adapt messaging for their target audiences, a process that is often a challenge for local organizations (46). Additionally, local organizations may provide other wrap-around services to create an environment that enables low SEP individuals to take advantage of the information contained in GHWs and associated campaigns (47). This is no easy task, particularly when promoting tobacco control among low SEP individuals who are already facing high levels of competing demands. Local organizations, with their tremendous reach, particularly among populations that are often hard to reach by traditional channels, may have a unique advantage here (48).

Taken together, the themes that emerged from this study highlight the potential to activate local networks in support of tobacco control through GHWs. However, given the limited funds available for such activities, it will be critical to consider redirecting or consolidating existing efforts. These types of solutions can leverage existing financial and staffing resources and can also leverage existing networks held by the third parties identified as important supports (such as the state department of public health). Importantly, the findings prompt consideration of wrap-around local health promotion activities for policy implementation in the same way one might for a mass media campaign. If there is an external force driving prevention and cessation activities, local organizations should be positioned to make the most of it and to ensure that benefits accrue to underserved populations. As described by the Communication Inequalities theoretical perspective, such supports will be critical to address potential individual- and group/institutional- level inequalities that could be generated by the implementation of GHWs nationally.

As with any study, the findings must be placed in the context of a set of limitations. First, participants were made aware of GHWs and were presented with images of the potential GHWs, but would not necessarily have full knowledge of the accompanying rules and regulations, so may not have been able to anticipate the effects fully. Second, the three communities under study were all in Massachusetts, which has a progressive tobacco control system; thus the findings may not be applicable to states and regions with a markedly different context. At the same time, the study has several strengths that outweigh the limitations. First, we conducted the study in three diverse communities and with participants from a wide range of sectors that may be engaged in support of GHW implementation. Second, we focused on organizations and individuals that engage in health outreach among low SEP individuals, a group that is experiencing tobacco-related disparities and often experiences communication inequalities as well.

Overall, the study points to the potential for local organizations to play an active and important role in supporting the implementation of GHWs. Engaging stakeholders early and in an ongoing manner, along with provision of necessary supports, can leverage existing local resources with important impacts for tobacco control and tobacco-related disparities.

## Ethics statement

This study was carried out in accordance with the recommendations of the Institutional Review Board of the Harvard T.H. Chan School of Public Health, with written informed consent from all subjects. All subjects gave written informed consent in accordance with the Declaration of Helsinki. The protocol was approved by the Institutional Review Board of the Harvard T.H. Chan School of Public Health.

## Author contributions

KV conceptualized and received funding for the study. SR and KV planned the analysis. SR and JA-M analyzed the data. SR drafted the manuscript. SR, JA-M, RN, and KV revised the manuscript and approved the final version.

### Conflict of interest statement

The authors declare that the research was conducted in the absence of any commercial or financial relationships that could be construed as a potential conflict of interest.
